# The Study of the Transient Dose Rate Effect on ROIC Pixels in Ultra-Large-Scale Infrared Detectors

**DOI:** 10.3390/mi16060700

**Published:** 2025-06-12

**Authors:** Yuan Liu, Bin Wang, Ziyuan Tang, Mengwei Chen, Hui Wang, Weitao Yang, Longsheng Wu

**Affiliations:** State Key Laboratory of Wide Bandgap Semiconductor Technology, Faculty of Integrated Circuit, Xidian University, Xi’an 710071, China

**Keywords:** N-well, transient dose rate effect, pixel, TCAD

## Abstract

Infrared image sensors are crucial across various industries. However, with technological advancements, the growing scale of infrared image sensors has made the impact of transient dose rate effects increasingly significant. It is necessary to conduct relevant radiation effect studies to provide the theoretical and data basis for future radiation-hardened design. This study explores the response of large-area N-wells in the readout circuit of infrared detectors to transient dose rate effects. The TCAD simulation results indicate that the expansive N-well area in the merged-design pixel units generates significant current pulses when exposed to gamma-ray irradiation. Specifically, at dose rates of 3 × 10^11^ rad/s, 5 × 10^11^ rad/s, 7 × 10^11^ rad/s, and 9 × 10^11^ rad/s, the pulse currents measured are 39 nA, 64 nA, 89 nA, and 119 nA, respectively. Due to the spatial constraints of the 55 nm merged design, the close proximity of the GND to the N-well creates a high potential barrier near the N-well, obstructing the path between the GND and the substrate, which results in the pulse current exhibiting a stepped-like characteristic.

## 1. Introduction

Transient dose rate effect (TDRE) refers to the transient response or damage caused by the exposure of electronic devices or materials to high-intensity ionizing radiation over extremely short timescales (nanosecond to microsecond ranges), where the instantaneous high dose rate (radiation dose per unit time) induces rapid ionization effects. This phenomenon is commonly observed in extreme radiation environments such as nuclear explosions (the instantaneous dose rate can reach up to 10^11^ rad/s) [1], potentially leading to electronic system malfunctions or even permanent damage. For deep sub-micron CMOS integrated circuits, the transient dose rate effect is the most complex and challenging to harden among various radiation effects [2]. To date, significant research has been conducted within academia on the response of LDOs [3], comparators [4], and commercial FPGAs to transient dose rate effects [5]. Simultaneously, there are teams that have researched the transient radiation effects on RF integrated circuits [6] and GaAs-buffered FET logic integrated circuits [7].

Infrared image sensor readout circuits are special, large-scale, high-repeatability integrated circuits. As a key part of infrared image sensors, they enable sensor applications in medicine, military fields, etc. As technology advances, the array scale of these sensors is continually expanding. In June 2024, Sony Semiconductor introduced a new global shutter CMOS image sensor, the “IMX901” [8], which boasts an 8 K horizontal resolution, a 2 K vertical resolution, and approximately 16.4 million effective pixels. At such large array scales, the impact of transient dose rate effects has become increasingly significant.

However, the existing studies are all on the total dose effect and displacement damage effect of pixels. There is little study on the transient dose rate effect of pixels in infrared detectors. The Goiffon team has compared the density of interface states and trapped charge density to study the source of total dose-induced dark current in CIS (CMOS Image Sensor) and confirmed with TCAD simulation that radiation-induced trapped charges expand the depletion region of the oxide interface, thereby increasing the SRH generation current of interface states [9,10,11,12,13]. Chen et al. investigated the characteristics of the 5T PPD CIS pixel structure with an optimized row selector under X-ray irradiation. They found that X-ray irradiation increases acceptor-like interface traps and enhances the dark random noise of the BSF pixel, thereby reducing its sensitivity to radiation [14]. Wang Zujun’s team has employed TCAD software (Sentaurus 2013) to simulate the radiation effects on CIS, leveraging irradiation experimental results. Their simulations and analyses have focused on elucidating the degradation mechanisms of dark current and image retention within 4T PPD CIS pixel units under total dose irradiation conditions [15]. Additionally, Wang Fan and Li Yudong, along with their colleagues, have designed experiments involving total ionizing dose irradiation. They have further explored the physical underpinnings of how total dose influences the full-well capacity and dark current characteristics in 4T PPD pixels of CIS [16]. The above research mainly focuses on the functional degradation of CMOS image sensors caused by the irradiation effects of visible—light pixel circuits [11,17,18,19,20,21,22,23,24,25,26,27,28,29]. In recent years, significant progress has also been made in the research of infrared image sensors. In 2019, Yoshiharu Ajiki and his team reported an infrared photodetector (IR-PD). It was based on organic crystal materials and plasmonic nano—pillar structures. The detector was capable of detecting infrared light beyond 2.0 μm [30]. In 2021, Wang et al. discussed emerging single-photon detectors based on low-dimensional materials, discussing their working mechanisms, performance metrics, and challenges, while highlighting their potential for next-generation photon-counting technologies [31]. In 2024, Vallone et al. employed TCAD simulations and theoretical analysis to investigate the optimal doping concentration for minimizing dark current in HgCdTe infrared barrier detectors, providing valuable design insights for detector development [32]. In 2024, Hailu Wang et al. reported the observation of a low-threshold avalanche effect in a stepwise WSe_2_ homojunction. Enabled by weak electron-phonon scattering and high electric fields, this room-temperature effect significantly reduces the threshold voltage and exhibits high sensitivity with low dark current, holding promise for future photovoltaic and optoelectronic devices [33]. The above-mentioned research on infrared detectors mainly focuses on designing and optimizing new high-performance pixels, while studies on the radiation effects of readout circuits, particularly the transient dose—rate effects of ROIC (Read-out Integrated Circuit) pixels, are relatively rare. Additionally, driven by the requirements for pixel output swing and various non-ideal factors, the size of pixels in readout circuits for infrared image sensors is shrinking, leading to the emergence of merged designs for multiple pixels [34,35,36]. In such merged pixel designs, PMOS transistors share a common N-well to minimize area loss, a configuration that relies on the spacing between multiple N-wells. Notably, the enlarged N-well area exhibits heightened sensitivity to transient dose-rate effects.

Therefore, this study investigates the transient dose rate response of large-area N-well structures in existing image sensors. The result reveals that in gamma-irradiated ultra-large-scale infrared image sensor pixel units with merged architecture, a transient photocurrent pulse is generated upon irradiation. Notably, the amplitude of the transient photocurrent pulse increases proportionally with the dose rate. However, owing to the constrained dimensions of the ultra-large-scale pixel unit, the spatial proximity between the substrate grounding point and the N-well induces significant overlap between the depletion region at the N-well/substrate interface and the substrate contact. This geometric configuration results in a distinctive “stepped-like” characteristic in the transient photocurrent waveform rather than exhibiting conventional pulse behavior. Meanwhile, radiation-induced transient currents impose stringent requirements on the current-carrying capacity of the power bus in pixel arrays. Excessive transient dose rates would generate correspondingly large instantaneous currents, potentially leading to circuit burnout.

## 2. Simulation Details

**Figure 1:**
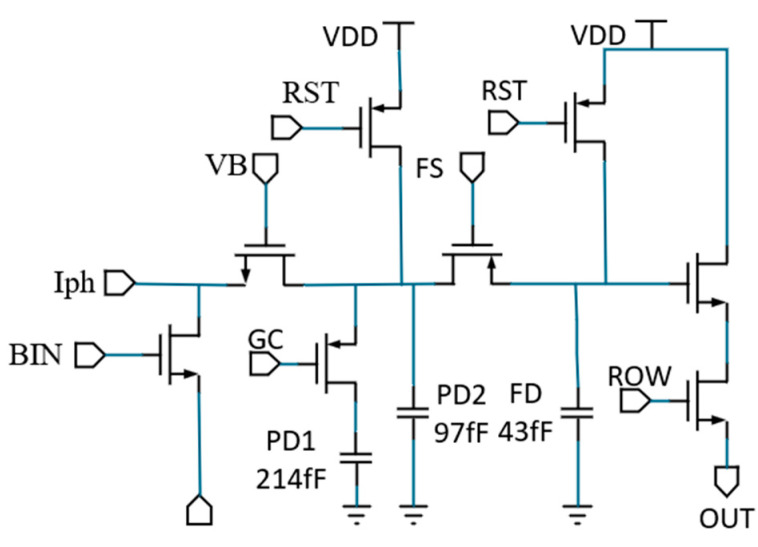
Schematic diagram.

This investigation centers on a merged-design ultra-large-scale infrared image sensor pixel architecture with a pixel array size of 8 K × 8 K. As depicted in Figure 1, the circuitry consists of eight transistors, comprising four NMOS and four PMOS transistors. In the design, considering factors like the body effect, the GC transistors are PMOS, and PMOS is employed in the FS transistor to isolate the FD node from substrate noise. Also, the RST transistor, which resets the circuit to V_DD_, is designed with a PMOS to increase the swing. During operation, the reset transistor is initially activated to reset both the photodiode (PD) node and the floating diffusion (FD) node. Next, the transfer gate (TX) transistor is turned on to transfer the photogenerated charge from the PD node to the storage region. After this, a secondary reset of the FD node is performed. Subsequently, the floating diffusion switch (FS) transistor is enabled to transfer the stored charge from the PD node to the FD node for readout through the source follower. Once the charge has been transferred to the FD node, the PD node is reset again and remains idle in preparation for the next exposure cycle.

The pixel circuit features dual full-well capacity (FWC) configurations of 1 Me^−^ and 3 Me^−^, achieved through switchable capacitor integration. The gate control (GC) signal determines the selection of FWC: (1) In GC-off mode, single-capacitor coupling to the photodiode (PD) node results in a FWC of 1 Me^−^. (2) With GC-on activation, parallel capacitor integration is enabled, which increases the FWC to 3 Me^−^, thus enhancing dynamic range adaptation.

**Figure 2:**
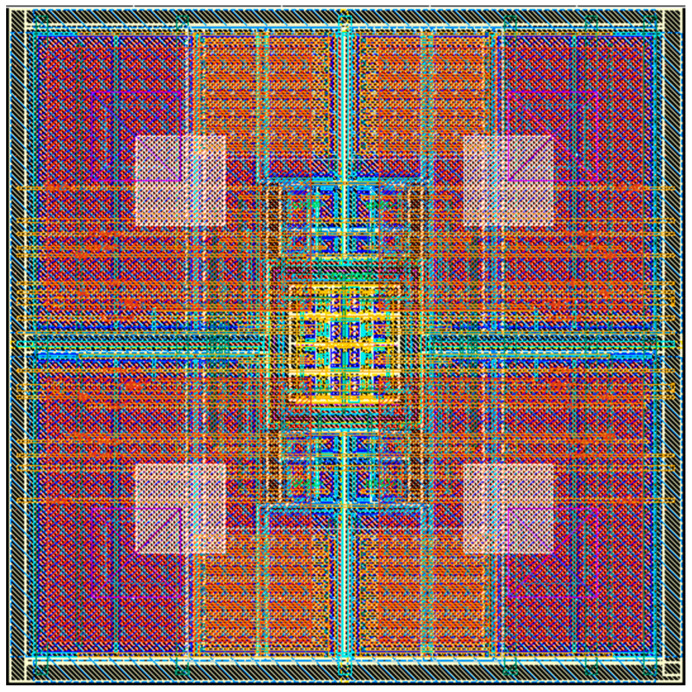
Circuit layout (four pixel units with a merged design).

**Figure 3:**
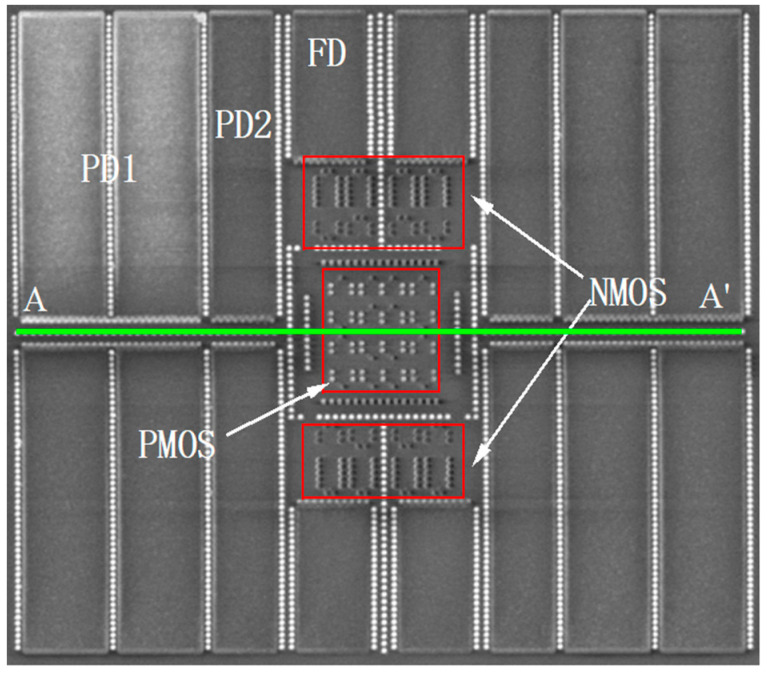
Photograph of the physical device (four pixel units with a merged design).

Figure 2 and Figure 3 present the layout design and the physical photograph of the study subject, respectively. Given a unit capacitance constraint of 4.37 fF/μm^2^, achieving a 3 MeV full-well capacity necessitates that the capacitance area per pixel exceeds 76 square micrometers. However, due to design specifications, the PMOS devices within the pixel cannot fulfill the area constraint of 10 μm × 10 μm, even when sharing an N-well. Consequently, a 2 × 2 pixel merged design was implemented, allowing the PMOS transistors of four pixels to share the N-well and meet the full-well capacity requirements. Significantly, to maintain a compact design, the 2 × 2 pixel configuration features a large N-well, with an area exceeding 5 μm × 5 μm.

**Figure 4:**
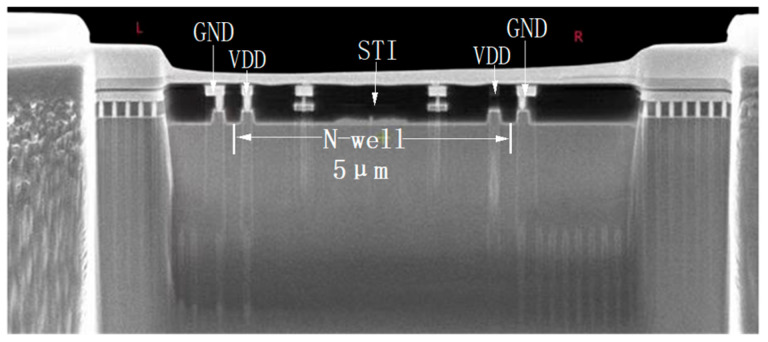
N-well cross-section photo.

Figure 4 presents the lateral cross-sectional view of the circuit used in the study, focusing on the N-well. The cross-section is taken at the A-A’ line in Figure 3. The N-well has a depth of 1 μm and a width of 5 μm to accommodate all PMOS transistors of the four pixel units. The shallow trench isolation, with a depth of 250 nm, effectively isolates different doped regions. Due to layout area constraints and the merged design of the four pixel units, the distance between GND and the N-well is significantly reduced to 170 nm.

**Figure 5:**
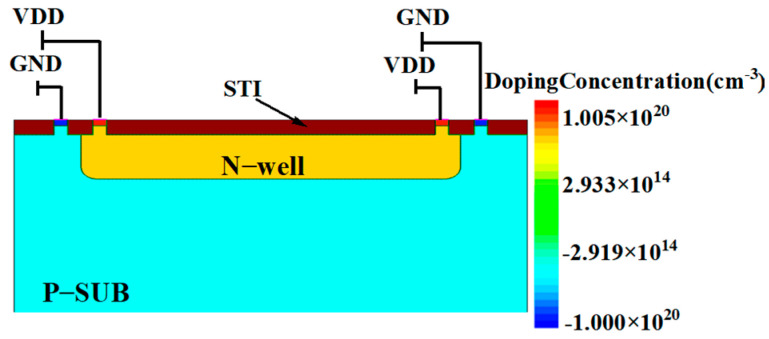
Simulation model.

For the N-well area, which is the focus of the study, a model was created in the TCAD simulation software as shown in Figure 5. The relevant parameters are shown in Table 1.

The simulation model adopted the SRH recombination model, mobility model (including PhuMob, HighFieldSaturation, Enormal), Hydrodynamic model, EffectiveIntrinsicDensity model, and radiation simulation through the Radiation model [6].

## 3. Simulation Results and Discussion

**Figure 6:**
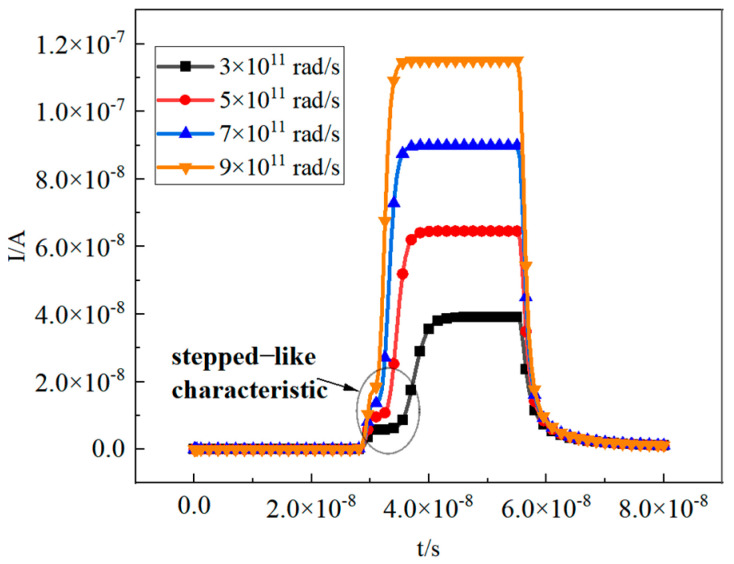
Pulse currents in the N−well are generated at different dose rates (V_DD_ = 3 V).

Figure 6 shows the instantaneous current of the N-well when the dose rate is set to 3 × 10^11^ rad/s, 5 × 10^11^ rad/s, 7 × 10^11^ rad/s, and 9 × 10^11^ rad/s. The results show that as the dose rate increases, the pulse currents are 39 nA, 64 nA, 89 nA, and 119 nA, respectively. For the merged-design pixel array, the simulation defaults to an N-well width of 1 μm, whereas the actual value is 5 μm. Measured transient currents under irradiation are 0.78 A, 1.28 A, 1.78 A, and 2.38 A. With the power bus capacity limited to 1 mA/μm, these current levels under excessive dose rates directly exceed design specifications, causing circuit burnout.

Additionally, the instantaneous light current exhibits a stepped-like characteristic as irradiation proceeds, with larger transient dose rates corresponding to shorter stages and higher peak amplitude of the instantaneous pulse current. The core mechanism underlying the dose rate-induced increase in pulse current lies in the linear growth of energy deposited per unit time and the stable charge-collection efficiency. As the γ-ray dose rate increases, more high-energy photons enter the PN-junction (N-well-Psub) depletion layer per unit time. These photons transfer energy to secondary electrons via mechanisms like the photoelectric effect and Compton scattering. The number of electron–hole pairs (EHPs) generated during ionization then increases linearly. Since the strong electric field maintained by the reverse-bias voltage (VDD) remains stable within the depletion layer (with a charge-collection efficiency of nearly 100%), the effective charge collected by the electrodes per unit time (i.e., current I = ΔQ/Δt) also increases accordingly. This directly boosts the peak amplitude of the instantaneous pulse current.

**Figure 7:**
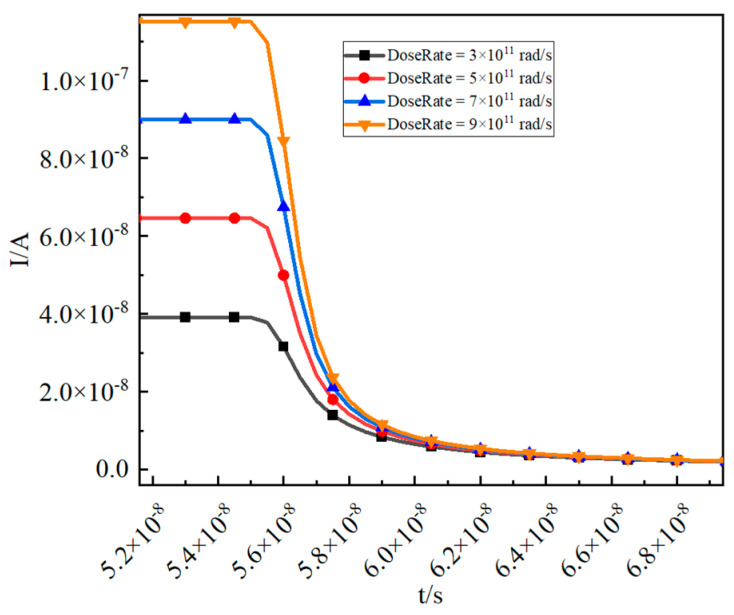
As the photogenerated carriers recombine, the tail current gradually diminishes.

As shown in Figure 6, a tail current can be detected immediately after irradiation ends, gradually diminishing as the photogenerated carriers recombine. Figure 7 demonstrates that irradiation ceases at 55 ns, resulting in a tail current that effectively disappears by 66 ns.

**Figure 8:**
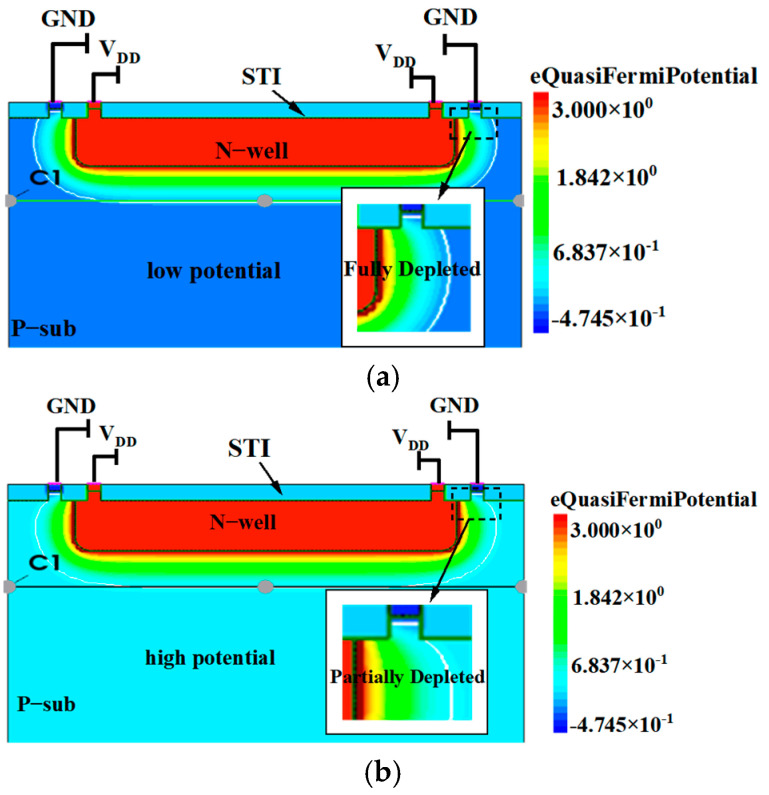
Potential distribution of the device at the beginning of 1.5 ns and after 20 ns irradiation: (**a**) 1.5 ns, (**b**) 20 ns.

Figure 8a illustrates the potential distribution within the device at 1.5 ns and 20 ns following the initiation of irradiation, corresponding to the “low pulse” and “high pulse” of the current pulse, respectively, with an irradiation dose rate of 3 × 10^11^ rad/s. The figure reveals a potential barrier at the interface between the bottom of the N-well and the substrate. Consequently, the holes generated in the substrate struggle to overcome this potential barrier to be collected by the electric field, resulting in a relatively low pulse current at this time. However, as irradiation continues, holes begin to accumulate in the substrate, leading to an increase in potential, as depicted in Figure 8b. At this stage, the holes can be effectively collected by the electric field, thereby resulting in an increase in pulse current.

**Figure 9:**
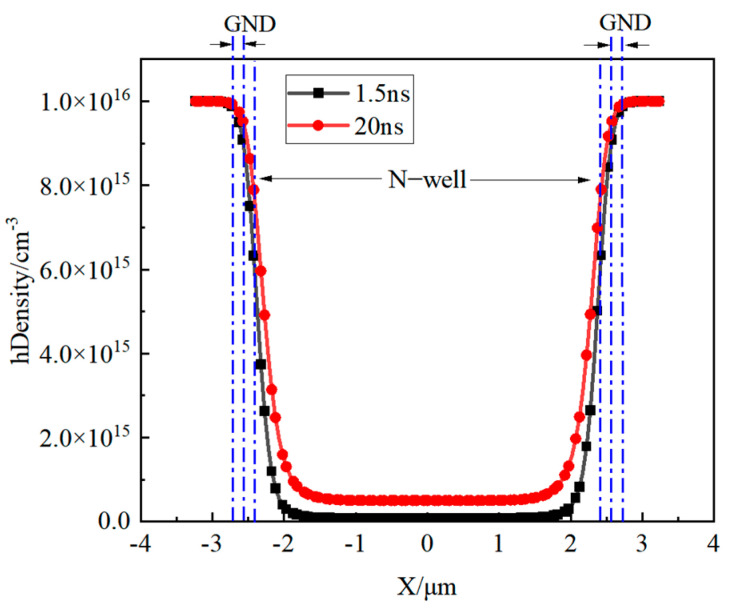
Hole distribution at C1 location at irradiation times of 1.5 ns and 20 ns (DoseRate = 3 × 10^11^ rad/s).

To assess the hole concentration at location C1, Figure 9 presents the hole concentration in the substrate at both 1.5 ns and 20 ns after the initiation of irradiation, demonstrating that the hole concentration in the substrate rises with the progression of the irradiation process.

**Figure 10:**
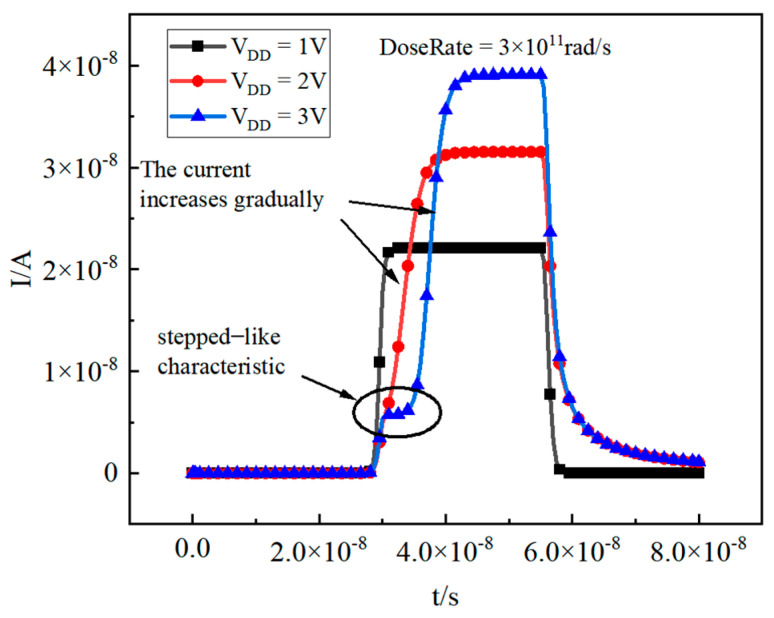
Pulse current when V_DD_ is set to different values (DoseRate = 3 × 10^11^ rad/s).

The simulation model remains consistent, but the N-well potential is adjusted to 1 V, 2 V, and 3 V, with simulations conducted accordingly. The results are illustrated in Figure 10. At V_DD_ levels of 1 V and 2 V, the pulse current does not demonstrate a step-like characteristic. Additionally, when V_DD_ is set to 2 V, the rising edge of the pulse is noticeably slower compared to when V_DD_ is at 1 V. This delay occurs because, at 2 V, the depletion region expands, creating a narrower path for GND to collect holes, which in turn slows the rising edge. However, at V_DD_ of 3 V, the step-like characteristic re-emerges. Additionally, it can be observed that as the voltage increases, the peak value of the pulse current correspondingly increases. When gamma rays pass through the substrate, they generate a large number of electron–hole pairs (EHPs). A higher VDD leads to a wider depletion region between the N-well and the substrate. This expanded depletion region improves the collection efficiency of radiation-generated EHPs. In addition, a higher VDD enhances electron mobility within the substrate and reduces the likelihood of electron–hole recombination during carrier transit. These two mechanisms work together to increase the peak photocurrent magnitude.

**Figure 11:**
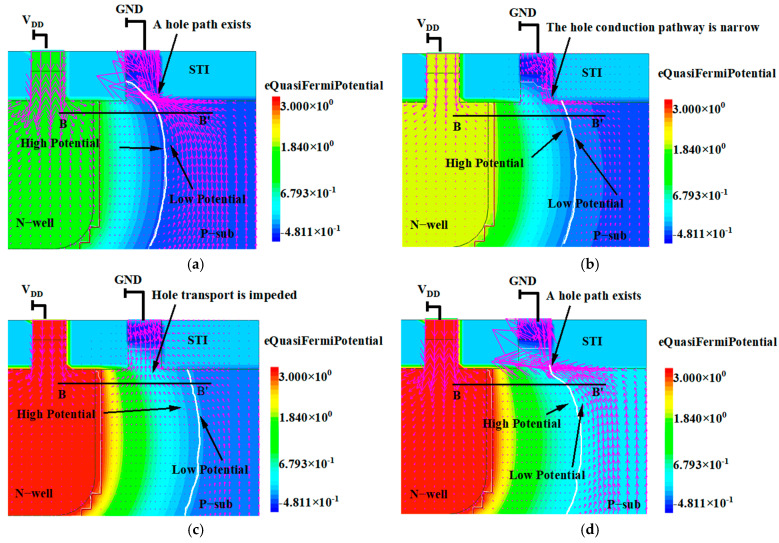
Potential distribution under different V_DD_: (**a**) V_DD_ = 1 V, dose time = 1.5 ns; (**b**) V_DD_ = 2 V, dose time = 1.5 ns; (**c**) V_DD_ = 3 V, dose time = 1.5 ns; (**d**) V_DD_ = 3 V, dose time = 20 ns.The direction of the purple arrows indicates the direction of the current, while the density of the arrows indicates the current density.

Figure 11 illustrates the potential distribution 1.5 ns after the initiation of irradiation at V_DD_ levels of 1 V, 2 V, and 3 V, as well as the potential distribution at 3 V after 20 ns of irradiation. The density of the pink arrows represents the current density. In these figures, the white lines indicate the boundaries of the depletion regions. The simulation results reveal that, with V_DD_ set to 1 V and after 1.5 ns of irradiation, the width of the depletion region in the substrate measures 0.305 μm. For the other three scenarios, the widths are 0.39 μm, 0.47 μm, and 0.35 μm, respectively. These variations in width lead to differences in the high-potential barrier widths. It is evident from the figures that at V_DD_ of 1 V, the depletion region is the narrowest, allowing for the widest path for GND to collect holes. Consequently, the rising edge of the pulse current is the steepest, and no step-like characteristic occurs. At V_DD_ of 2 V, the path becomes slightly narrower; although there is still no stage in the pulse current, the rise is more gradual. However, at V_DD_ of 3 V, the path is effectively nonexistent; however, as holes accumulate, the conduction pathway reopens, leading to an increase in the pulse current, resulting in the presence of a step-like characteristic in the pulse current.

**Figure 12:**
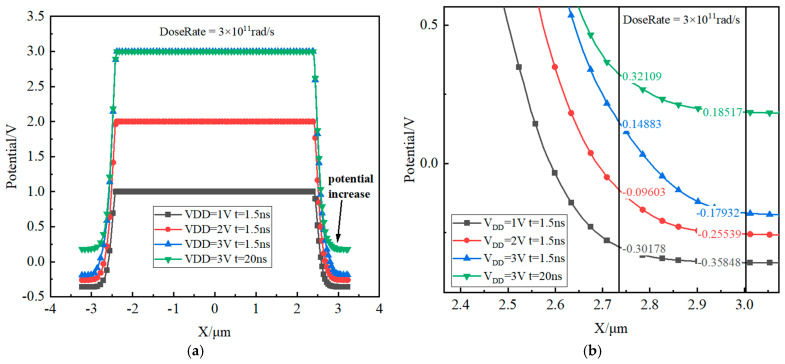
Potential distribution under different V_DD_ conditions (DoseRate = 3 × 10^11^ rad/s). (**a**) Overall and (**b**) local magnification.

Figure 12 shows the potential distribution curve along the B-B’ direction in Figure 11. In the figure, the X-axis coordinate of the right edge of the substrate is X = 2.73 μm.

When V_DD_ is set to 1 V with an irradiation duration of 1.5 ns, it is observed that the ∆V (the potential difference between the right edge of the substrate contact and the substrate) = 0.0567 V. Under these conditions, the potential barrier remains low, facilitating efficient hole collection by the substrate contact, as illustrated by the black curve in Figure 10. Increasing V_DD_ to 2 V with the same irradiation duration (1.5 ns) results in a rise in ∆V to 0.15963 V. At this level, the relatively higher potential barrier allows for only a gradual collection of holes, as indicated by the red curve in Figure 10. At V_DD_ = 3 V and 1.5 ns of irradiation, ∆V increases to 0.32815 V, where the significantly heightened potential barrier greatly hinders hole collection, leading to a reduced current. However, when the irradiation time is extended to 20 ns at V_DD_ = 3 V, ∆V decreases to 0.13592 V. This reduction in the potential barrier facilitates progressive hole collection until steady-state conditions are achieved, as depicted by the green curve in Figure 10.

This phenomenon can be controlled or optimized to some degree, and the specific approach depends on the operating mode of the circuit. In multi-frame continuous acquisition mode, where the circuit operates for extended periods, VDD can be strategically reduced. This reduction promotes efficient collection of radiation-generated electron–hole pairs, preventing excessive localized noise caused by charge accumulation within the substrate. However, during single-frame acquisition operation, VDD can be maintained at 3.3 V. In this mode, the readout circuitry operates only for a short time before being powered down. Since the photocurrent pulse remains in its suppressed (low-magnitude) phase during this brief active period, it minimizes the transient photocurrent magnitude and thus protects the circuitry.

## 4. Conclusions

The extended N-well geometry enhances the generation of transient current, which in turn compromises the circuit’s transient dose rate hardness. Elevated transient dose rates will induce circuit burnout. The pulse current displays a stepped-like characteristic. This multistage current behavior arises from the dynamic modulation of the potential barrier at the N-well/substrate interface. Initially, the inherent potential barrier obstructs efficient hole collection across the junction. However, as irradiation continues, the accumulation of holes in the substrate raises the local potential, subsequently reducing the effective barrier height. This potential-dependent transport mechanism facilitates progressive hole collection, resulting in distinct step-like increments in photocurrent amplitude during prolonged irradiation.

## Figures and Tables

**Table 1:** Parameters of the simulation model.

Category	Parameter	Value
Process Technology	MOSFET Technology Node	55 nm
Device Geometry	NMOS W/L Ratio	380 nm/400 nm
	PMOS W/L Ratio	330 nm/400 nm
Isolation Structure	STI Depth	250 nm
N-well Profile	Depth	1 μm
Gate Oxide	Material	SiO_2_
	Thickness	7 nm
Doping Concentration	Substrate (Boron)	1 × 10^16^ cm^−3^
	N^−^ Well (Phosphorus)	5 × 10^17^ cm^−3^
	P^+^ Region (Boron)	1 × 10^20^ cm^−3^
	N^+^ Region (Phosphorus)	1 × 10^20^ cm^−3^
	Source/Drain	1 × 10^20^ cm^−3^
Electrical Characteristics	Operating Voltage	3.3 V

## Data Availability

The original contributions presented in this study are included in the article. Further inquiries can be directed to the corresponding author.

## References

[1] Cai X. (2022). Nuclear Explosion Effects and Protection.

[2] Wang Z., Xue Y., Liu M., Xu R., Ning H., Gao W. (2019). Transient Response in PPD CMOS Image Sensors Irradiated by Gamma Rays: Variation of Dose Rates and Integration Times. IEEE Trans. Nucl. Sci..

[3] Li R., Li J., Wang C., He C. Transient radiation effects in several types of LDO. Proceedings of the 2021 4th International Conference on Radiation Effects of Electronic Devices (ICREED).

[4] Li J., Li Y., Guo Y., Li R., Chen W., Liu Y. (2023). Investigation of Transient Dose-Rate Effect on High-Speed Comparator SB9696. IEEE Trans. Nucl. Sci..

[5] Li Y., Li J., Tang G., Li R., He C., Xiao Y., Chen W., Li Y., Guo Y., Zhang G. (2025). Pulsed-Laser Testing to Evaluate Transient Dose Rate Effect on a Commercial-off-the-Shelf FPGA. IEEE Trans. Nucl. Sci..

[6] Nikiforov A.Y., Elesin V.V., Chukov G.V., Amburkin D.M., Nazarova G.N. Long-Term Transient Radiation Effects in SOI CMOS RF ICs. Proceedings of the 2015 15th European Conference on Radiation and Its Effects on Components and Systems (RADECS).

[7] Anderson W.T., Simons M., Forbes L., Koyama R.Y., Reeder T.M. Transient Radiation Upset of GaAs Buffered FET Logic ICs. Proceedings of the 1987 IEEE GaAs IC Symposium Technical Digest.

[8] Sony Semiconductor Solutions Group Wide High-Resolution, Global Shutter Image Sensor with Horizontal 8K/6K Resolution, 1.4-inch Size and Approx. 16.41 Million Pixels IMX901, 1.1 -inch Size and Approx. 12.38 Million Pixels IMX902 | Products & Solutions | Sony Semiconductor Solutions Group. https://www.sony-semicon.com/cn/products/is/industry/gs/imx901-902.html.

[9] Goiffon V., Virmontois C., Magnan P., Girard S., Paillet P. (2010). Analysis of total dose—Induced dark current in CMOS image sensors from interface state and trapped charge density measurements. IEEE Trans. Nucl. Sci..

[10] Goiffon V., Estribeau M., Marcelot O., Cervantes P., Magnan P., Gaillardin M. (2013). Radiation Effects in Pinned Photodiode CMOS Image Sensors: Pixel Performance Degradation Due to Total Ionizing Dose. IEEE Trans. Nucl. Sci..

[11] Place S., Carrere J.-P., Allegret S., Magnan P., Goiffon V., Roy F. (2012). Radiation effects on CMOS image sensors with sub-2 pinned photodiodes. IEEE Trans. Nucl. Sci..

[12] Marcelot O., Goiffon V., Rizzolo S., Pace F., Magnan P. (2017). Dark current sharing and cancellation mechanisms in CMOS image sensors analyzed by TCAD simulations. IEEE Trans. Electron Devices.

[13] Marcelot O., Goiffon V., Magnan P. (2019). Exploration of pinned photodiode radiation hardening solutions through TCAD simulations. IEEE Trans. Electron Devices.

[14] Chen Y., Tan J., Wang X., Mierop A.J., Theuwissen A.J.P. In-pixel buried-channel source follower in CMOS image sensors exposed to X-ray radiation. Proceedings of the SENSORS, 2010 IEEE.

[15] Wang Z., Liu C., Ma Y., Wu Z., Wang Y., Tang B. (2015). Degradation of CMOS APS image sensors induced by total ionizing dose radiation at different dose rates and biased conditions. IEEE Trans. Nucl. Sci..

[16] Wang F., Li Y.-D., Guo Q., Wang B., Zhang X.-Y., Wen L., He C.-F. (2016). Total ionizing dose radiation effects in foue-transistor complementary metal oxide semiconductor image sensors. Acta Phys. Sin..

[17] Hopkinson G.R. (2000). Radiation effects in a CMOS active pixel sensor. IEEE Trans. Nucl. Sci..

[18] Cohen M., David J.-P. (2000). Radiation-induced dark current in CMOS active pixel sensors. IEEE Trans. Nucl. Sci..

[19] Eid E.-S., Chan T.Y., Fossum E.R., Tsai R.H., Spagnuolo R., Deily J., Byers W.B., Peden J.C. (2001). Design and characterization of ionizing radiation-tolerant CMOS APS image sensors up to 30 Mrd(Si)total dose. IEEE Trans. Nucl. Sci..

[20] Hancock B.R., Cunningham T.J., McCarty K.P., Yang G., Wrigley C.J., Ringold P.G., Stirbl R.C., Pain B. Multi-megarad(Si) radiation-tolerant integrated CMOS imager. Proceedings of the SPIE 4306, Sensors and Camera Systems for Scientific, Industrial, and Digital Photography Applications II.

[21] Bogaerts J., Dierickx B., Meynants G., Uwaerts D. (2003). Total dose and displacement damage effects in a radiation-hardened CMOS APS. IEEE Trans. Electron Devices.

[22] Pain B., Hancock B.R., Cunningham T.J., Seshadri S., Sun C., Pedadda P., Wrigley C.J., Stirbl R.C. Hardening CMOS imagers: Radhard-by-design or radhard-by-foundry. Proceedings of the SPIE 5167, Focal Plane Arrays for Space Telescopes.

[23] Beaumel M., Hervé D., Van Aken D. Cobalt-60, proton and electron irradiation of a radiation-hardened active pixel sensor. Proceedings of the 2009 European Conference on Radiation and Its Effects on Components and Systems.

[24] Goiffon V., Estribeau M., Cervantes P., Molina R., Gaillardin M., Magnan P. (2014). Influence of transfer gate design and bias on the radiation hardness of pinned photodiode CMOS image sensors. IEEE Trans. Nucl. Sci..

[25] Innocent M. A radiation tolerant 4t pixel for space applications: Layout and process optimization. Proceedings of the 2013 International Image Sensor Workshop.

[26] Qian X., Yu H., Chen S., Low K.S. Design and characterization of radiation-tolerant CMOS 4T Active Pixel Sensors. Proceedings of the 2014 International Symposium on Integrated Circuits (ISIC).

[27] Rao P.R., Wang X., Theuwissen A.J.P. Degradation of CMOS image sensors in deep-submicron technology due to-irradiation. Proceedings of the 37th European Solid State Device Research Conference.

[28] Chen Y., Tan J., Wang X., Mierop A.J., Theuwissen A.J.P. X-ray radiation effect on CMOS imagers with in-pixel buried-channel source follower. Proceedings of the European Solid-State Device Research Conference (ESSDERC).

[29] Wang X., Bogaerts J., Ogiers W., Beeckman G., Meynants G. Design and characterization of radiation tolerant CMOS image sensor for space applications. Proceedings of the SPIE 8194, International Symposium on Photoelectronic Detection and Imaging 2011: Advances in Imaging Detectors and Applications.

[30] Ajiki Y., Kan T., Yahiro M., Hamada A., Adachi J., Adachi C. INFRARED DETECTOR USING ORGANIC NANO-PILLAR ARRAYS. Proceedings of the 2019 20th International Conference on Solid-State Sensors, Actuators and Microsystems & Eurosensors XXXIII (TRANSDUCERS & EUROSENSORS XXXIII).

[31] Wang H., Guo J., Miao J., Luo W., Gu Y., Xie R., Wang F., Zhang L., Wang P., Hu W. (2022). Emerging Single-Photon Detectors Based on Low-Dimensional Materials. Small.

[32] Vallone M., Alasio M.G.C., Tibaldi A., Bertazzi F., Hanna S., Wegmann A. (2024). Exploring Optimal Dark Current Design in HgCdTe Infrared Barrier Detectors: A TCAD and Semianalytic Investigation. IEEE Photonics J..

[33] Wang H., Xia H., Liu Y., Chen Y., Xie R., Wang Z., Wang P., Miao J., Wang F., Li T. (2024). Room-temperature low-threshold avalanche effect in stepwise van-der-Waals homojunction photodiodes. Nat. Commun..

[34] Zhang Q., Guo Z. An 8T global shutter pixel with extended output range for CMOS image sensor. Proceedings of the 2019 IEEE International Conference on Electron Devices and Solid-State Circuits (EDSSC).

[35] Takayanagi I., Mo Y., Ando H., Kawamura K., Yoshimura N., Kimura K., Otaka T., Matsuo S., Suzuki T., Brady F. A 600 × 600 pixel, 500 fps CMOS image sensor with a 4.4 μm pinned photodiode 5-transistor global shutter pixel. Proceedings of the International Image Sensor Workshop.

[36] Le Roch A., Virmontois C., Goiffon V., Tauziède L., Belloir J.-M., Durnez C. (2018). Radiation-Induced Defects in 8T-CMOS Global Shutter Image Sensor for Space Applications. IEEE Trans. Nucl. Sci..

